# Thymosin β4 stabilizes hypoxia induced brain microvascular endothelial cell dysfunction through S1PR1 dependent mechanisms

**DOI:** 10.1038/s41598-025-28435-2

**Published:** 2025-12-01

**Authors:** William G. Stewart, Christina D. Hejl, Rakeshwar S. Guleria, Sudhiranjan Gupta

**Affiliations:** Translational Science Core (Biomarkers & Genetics), VISN 17 Center of Excellence for Research on Returning War Veterans, Central Texas Veterans Health Care System, 4800 Memorial Drive (151C), Waco, TX 76711 USA

**Keywords:** Blood brain barrier, Thymosin β4, S1PR1, Diseases, Medical research, Neurology, Neuroscience

## Abstract

Acute ischemic injury causes impairment of blood brain barrier (BBB) permeability and is considered as secondary insult in the brain after traumatic brain injury (TBI). The mechanisms underlying these events are incomprehensible and therefore therapeutic opportunities are limited. Although drugs have been showing some promise in TBI outcome, the restoration of BBB damage remain elusive. Thymosin β4 (Tβ4) is a secreted 43 amino acid peptide showed beneficial outcome in cerebral ischemia or TBI, however, it’s role in hypoxia-induced BBB damage remains elusive. We hypothesize that Tβ4 protect hypoxia-induced BBB disruption via Sphingosine 1–phosphate receptor 1 (S1PR1) modulation. In the current study, we investigated the beneficial effects of Tβ4 in hypoxia induced gene expression of several tight junction proteins, S1PR1, endothelial cell permeability and tight junction dynamics in human brain microvascular endothelial cells (hBMVECs), one of the important cell types in the BBB integrity. The data suggests that pretreatment with Tβ4 reversed the hypoxia-induced damage of BBB components in hBMVECs. Furthermore, results identify S1PR1, a possible target for Tβ4. Inhibition of S1PR1 showed that Tβ4 failed to offer protection. Together, data provided evidence that S1PR1 is pivotal and Tβ4 can serve as a protective agent in BBB integrity and may offer a promising therapeutic target. In conclusion, we propose that depletion of S1PR1signaling is vital in hypoxia-induced BBB pathophysiology and Tβ4 may be tested as a potential treatment modality and warrant further investigation.

**Supplementary Information:**

The online version contains supplementary material available at 10.1038/s41598-025-28435-2.

## Introduction

The blood-brain barrier (BBB) is a molecular sheath between circulating blood and the central nervous system (CNS). As such, the BBB plays a protective role in CNS homeostasis by acting as a vascular gatekeeper, controlling solute movements from circulating blood into brain parenchyma^1,2^. The structure of BBB is complex and consists of brain microvascular endothelial cells (BMVEC), astrocyte end-feet, pericytes and the basal lamina^1,3^. Together, these comprise a neurovascular unit (NVU). A characteristic feature of the BBB sheath is its permeability which is primarily mediated by an array of tight junction (TJ) proteins located on BMVECs, namely zonula occludens (ZO), claudins, and occludins^1,3,4^. Therefore, a continuous layer of microvascular endothelial cells is pivotal to the integrity of the BBB. Several pathological conditions like local hypoxia/ischemia, traumatic brain injury, etc., can disrupt the BBB lining, causing leakage and initiate a sequela of pathophysiological changes such as inflammatory events, solute imbalance, infiltration of immune cells, oedema formation and introduction of unnecessary blood proteins into the brain^3,5^. Regulating BBB dynamics may be a potential strategy for treating CNS disorders.

Following a traumatic event, hypoxia is a frequent secondary insult, aggravating brain damage in acute ischemic-hypoxic injury^5,6^. Hypoxia is an impairment in oxygen delivery and known to cause BBB dysfunction^6^. However, the mechanisms that drive this disruption remain unclear. TJ proteins are known to play an essential role in preventing cellular infiltration (leucocytes) and maintain BBB integrity^6^. It is reported that the pathological process that takes place in a hypoxic microenvironment causes disarrangement of TJ proteins in BMVECs, which leads to a reduction of TJ protein levels and ultimately to BBB dysfunction^7^. Among the TJ proteins, claudin 5 is highly expressed in the BMVECs that regulate BBB permeability and is indispensable for proper barrier function^8,9^. However, the detailed role of TJ proteins under hypoxic conditions is understudied and warrants additional investigation.

Water transport is another critical component in BBB damage following ischemic injury as water is accumulated within the cells. Aquaporin 4 (AQ 4), a member of water channel protein in AQ family control water fluxes in the brain parenchyma^10^. There are conflicting study regarding AQ4 in brain edema and hypoxic ischemic brain injury and further AQ 4’s role in hypoxia condition is currently not studied well using BMVEC^11,12^. Brain endothelial cells also express various transporters to help solutes and molecules deliver to the BBB as they cannot pass through passively through the BBB^13,14^. One group of BBB transporters is ATP-binding cassette (ABC) transporters that include the breast cancer resistance protein (BCRP; coded by the gene *ABCG2*), an efflux transporter actively managing wide range of substrates and maintain brain homeostasis^15^. The role of BCRP is evolving and little is known in hypoxic brain using BMVEC as a model.

Thymosin-β 4 (Tβ4), a G-actin sequestering protein, modulates actin polymerization in cytoskeletal dynamics and is encoded by the TMSB4X gene on the X-chromosome^16,17^. Tβ4 is a 43-amino acid protein present in all cells except red blood cells^18^. It is a multifunctional and widely distributed peptide that contributes an essential role in many physiological and pathological processes such as promoting angiogenesis and proliferation, inhibiting apoptosis and inflammation, and plays a protective role in cardiac diseases^19–23^. Additionally, Tβ4 is suggested to have a protective role in neurological injury and stroke^24–27^. Importantly, Tβ4’s ubiquitous distribution in the CNS suggests its contribution in many neuro-cellular processes such as axonal pathfinding, neurite formation, proliferation, and survival^28^. Similarly, our recent findings showed that Tβ4 restored lipopolysaccharide (LPS)-induced BBB damage in BMVECs^29^. This finding led us to investigate whether hypoxia-induced BBB damage, another important phenomenon that happens during TBI, stroke, etc. can be rescued by Tβ4.

Sphingosine 1–phosphate (S1P), a pleiotropic lipid mediator, exerts its’ vascular function through five (S1P_1–5_) G protein-coupled receptors (GPCRs) and controls cell migration, adhesion, survival, and proliferation^30^. It has been reported that S1P and its receptor S1PR1 participates in neurovascular diseases and is also critical player in endothelial barrier function^31,32^. Furthermore, S1P_1_ regulates G_i_-dependent Rac activation, promotes cytoskeletal reorganization, and plays a role in adherens junction assembly which is required for vascular barrier function^31–34^. Despite the role of S1P signaling or S1P-S1PR1 axis in barrier function, its involvement in neurological disorders is recently acknowledged in many clinical conditions^35^. A recent report indicated that S1PR1 signaling is protective to ischemic stroke^36^. On the other hand, S1P-lyase (SGPL1) localizing to the endoplasmic reticulum can irreversibly cleave S1P, generating two non-sphingolipids, aldehyde hexadecanal and ethanolamine phosphate^37,38^. A new study using mice, with SGPL1 deficient brains, showed a connection between S1P metabolism and neuroinflammation by activating NLRP3 inflammasome in astrocytes^39^. However, S1PR1 and SGPL1’s role in BBB integrity under hypoxic conditions are currently undetermined.

Till date, the role of Tβ4 in hypoxia-induced BBB damage is unknown. Furthermore, the role of S1PR1, AQ4 or BCRP in hypoxic environment is currently obscured. Previously, Tβ4 was reported to contribute a critical role in diverse physiological process^19–23^, however, its’ role in hypoxia-induced BBB damage is emerging and yet to be fully identified. The role of the Tβ4-S1PR1 axis in hypoxic BBB damage has not been reported. In the present study, we hypothesize that Tβ4 may protect hypoxia-induced neurovascular remodeling in human BMVEC (hBMVEC) by restoring S1PR1/SGPL1 molecules. We aim to investigate the impact of initial and late hypoxia (oxygen concentration 1%) on hBMVEC dysfunction examining TJ proteins and S1PR1 signaling. We used hBMVEC, a primary cell type that lines the brain capillaries and forms a monolayer with TJ proteins to control fluid transport and prevent solutes and toxins from passing through freely. Furthermore, BMVEC interacts with other brain cells like astrocytes and glial cells to maintain the BBB integrity. This is the reason to use hBMVEC to understand BBB functional dynamics at disease perspective, and further to manipulate for therapeutic intervention.

## Materials and methods

### Cell culture and hypoxia treatment

Primary hBMVECs (passage 3, cat no. ACBRI 376) were purchased from Cell Systems, Kirkland, WA, USA, cultured per manufacturer instruction and in accordance with previously described procedures using Complete Classic Medium with Serum and CultureBoost™ (cat. no. 4Z0-500; Cell Systems, USA)^29^. Passage 9 were used for all experiments. For hypoxia studies, cells were first allowed to grow into confluency under normoxic conditions for two days, followed by exposure to hypoxia (oxygen concentration 1%) or continued culture under normoxic conditions for 48 h. The study utilized short-term (2 h) and long-term (24 h) hypoxia. For Tβ4 treatment, the cells were pretreated with Tβ4 (cat no. SRP 3324, Millipore Sigma, USA) at a concentration of 1 µg/mL for 2 h prior to switching the cells in hypoxic chamber at 37˚C for 2 h and 24 h, respectively. Three separate experimental groups were used for all gene, protein, and immunofluorescence analyses Control (2 h and 24 h), 2 h hypoxia, 24 h hypoxia, Tβ4 (2 h and 24 h), Tβ4 + 2 h hypoxia and Tβ4 + 24 h hypoxia. The dose of Tβ4 was based on previously published articles^29^. For VPC2309 (Cat no. sc-362817, Santa Cruz, USA), an inhibitor of S1PR1 treatment, the cells were pretreated with VPC2309 at a concentration of 50 nmol for 2 h prior to switching the cells in hypoxic chamber. The dose for VPC2309 was calibrated by western blotting and qRT-PCR of S1PR1 gene. (See suppl 1). The current work was carried under an approved research protocol which was reviewed and approved by the Central Texas Veterans Health Care System (CTVHCS; protocol #00711), Institutional Biosafety Committee (IBC), Institutional Review Board (IRB) and Research and Development Committee (R&DC).

### RNA isolation and quantitative real-time PCR (q-RT-PCR) analyses

Total RNAs from the hBMVECs were extracted using miRNEasy kit (Qiagen, Valencia, CA) as per the manufacturer’s instructions. For real-time RT-PCR, 100 ng of total RNAs were reverse transcribed to cDNA using cDNA synthesis kit (OriGene, Rockville, MD, USA) by following the manufacturer’s instructions. Quantitative PCR was performed as described previously^29,40^. Analysis of gene expression was evaluated by 2^(ΔΔCt)^ method. Each sample was run in triplicate, repeated in three separate sets of experiments and GAPDH was used as an internal loading control. The gene-specific primers used for the study were purchased from OriGene Technologies, Inc., USA. The following primer sequences were used: 5’-CCTGTGACATCCTCTTCAGAGC-3’ (forward) and.

5’-CACTTGCAGCAGGACATGATCC-3’ (reverse) for S1PR1 (cat no. HP205287),

5’-GAACACTGCCATGCTCGTCTGT-3’ (forward) and

5’-GATGAGGAAGCCTCCCAGACAA-3’ (reverse) for SGPL1 (cat no. HP207342),

5’-GTCCAGAATCTCGGAAAAGTGCC (forward),

5’-CTTTCAGCGCACCATACCAACC-3’ (reverse) for Tjp1 (cat no. HP206807),

5’-ATGGCAAAGTGAATGACAAGCGG-3’ (forward) and

5’- CTGTAACGAGGCTGCCTGAAGT-3’ (reverse) for Occludin (cat no. HP206202), 5’- ATGTGGCAGGTGACCGCCTTC-3’ and.

5’- CGAGTCGTACACTTTGCACTGC-3’ for Claudin5 (cat no. HP206822),

5’- GATTCTGTGCCCACAGTAAGGC-3’ (forward) and

5’- TGGTCACAGAGCCACCTTCTTG-3’ (reverse) for VCAM1 (cat no. HP230503),

5’- GATTCCTACCGAGACTCTCCTC-3’ (forward) and

5’- TGGAAGGCATGGACACCGTCAT-3’ (reverse) for Nrg1 (cat no. HP228585),

5’- GTCTCCTCTGACTTCAACAGCG-3’ (forward) and

5’- ACCACCCTGTTGCTGTAGCCAA-3’ (reverse) for GAPDH (cat no. HP205798).

5’- GTTCTCAGCAGCTCTTCGGCTT-3’ (forward) and

5’- TCCTCCAGACACACCACGGATA-3’ (reverse) for BCRP (cat no. HP 208088).

5- GCCATCATTGGAGCAGGAATCC-3’ (forward) and

5’- ACTCAACCAGGAGACCATGACC-3’ (reverse) for AQ4 (cat no, HP205472).

### Immunofluorescence microscopy

The hBMVECs were seeded in 6-well coated cover glass and were treated as described previously^29^. In brief, cells were washed with 1X PBS, fixed, and blocked with 5 % BSA solution (Thermo Fischer Scientific, Inc., USA) kept for 30 min at room temperature and incubated with anti-S1PR1 antibody overnight at 4°C in antibody dilution buffer. The dilution of the above antibodies was at 1:400. The cells were then washed, incubated with corresponding secondary antibodies for 1-hour in dark conditions and mounted on the slide containing a drop of ProLong Gold anti-fade mounting media containing 4, 6-daimindo-2-phenylindole (DAPI). Fluorescence images at 40X magnification were captured by Leica DMi8 Imaging system. Anti-S1PR1 (cat. no. 63335, lot no.1) and DAPI (cat. no. 4083) were purchased from Cell Signaling Technology, Inc., USA.

### Western blotting

The protein lysates were prepared from three separate sets of experiments as described previously^29^. The cell lysates used were for determining the level of Claudin 5 (Cldn 5) was 15 mg, Occludin and S1PR1 were 10 mg per sample. In brief, A stain-free 10–15 % gradient Tris-Glycine eXtended™ gel (Bio-Rad Laboratories, Inc.) was used for protein separation. The protein was transferred onto a PVDF membrane using the Trans Blot Turbo transfer system (Bio-Rad Laboratories, Inc. USA) according to the manufacturer’s protocol. The membrane was blocking using EveryBlot Blocking Buffer (cat no. 12010020, Bio-Rad Laboratories, Inc.) for 5 min at room temperature. The images were taken using the ChemiDoc MP imaging system (Bio-Rad Laboratories, Inc. USA). Primary antibodies against Occludin (cat no. 91131, lot no. 2), claudin5 (cat no. 49564, lot no. 1), BRCP (ABCG2, cat no. 42078, lot no. 3), AQ 4 (cat no. 59678, lot no. 3) and S1PR1 (cat no. 63335, lot no. 1) were purchased from Cell Signaling Technology, Inc., Beverly, MA, USA. The dilution of the above antibodies was 1:1000, respectively. The immunoreactive bands were visualized using Clarity Max Western enhanced chemiluminescence (ECL) kit (cat. no. 1705062, Bio-Rad Laboratories, Inc., USA), before the density of the band was quantified and analyzed using the ImageJ 4.1) software (National Institutes of Health). We ran the parallel blots for GAPDH (cat no. 2118, loading controls) with the same concentration of protein as used for running Western blots for the target proteins.

### Permeability and trans-endothelial electrical resistance (TEER) assay

The permeability was performed using Endothelial Trans-well Permeability Assay Kit (cat no. CB6929 from Cell Biologics, Inc., USA) according to manufacturer’s instruction. Data expressed as A450 absorption readings are considered relative permeability. The BBB function was determined by TEER assay using Milicell ERS2 (Milipore, Massachusetts, USA) volt-ohm meter according to the manufacturer’s protocol. In brief, the hBMVEC, 1.5 × 10^6^ cells were seeded in gelatin-coated permeable polyester Transwell filter inserts (cat no. 3524, Corning Costar, Thermo Fischer Scientific, Inc. USA). The treatment for The TEER values was obtained by immersing the electrode so that the shorter tip is in the Millicell^®^ culture plate inserts, and the longer tip is in the outer well. The volt-ohm meter captured the transmembrane electrical resistance according to the current and was measured. A transwell without cells with only the medium was used as a blank control.

### Statistical analysis

Data are expressed as means ± Standard Error Mean (SEM) and were analyzed by student t-test or one-way analysis of variance (ANOVA) using Prism 5.0 Graph Pad software (Graph Pad, San Diego, CA, USA). A P-value less than 0.05 was considered statistically significant.

## Results

### Effects of Tβ4 on hypoxia-induced alteration of TJ gene expression and protein levels in hBMVECs

**Fig. 1:**
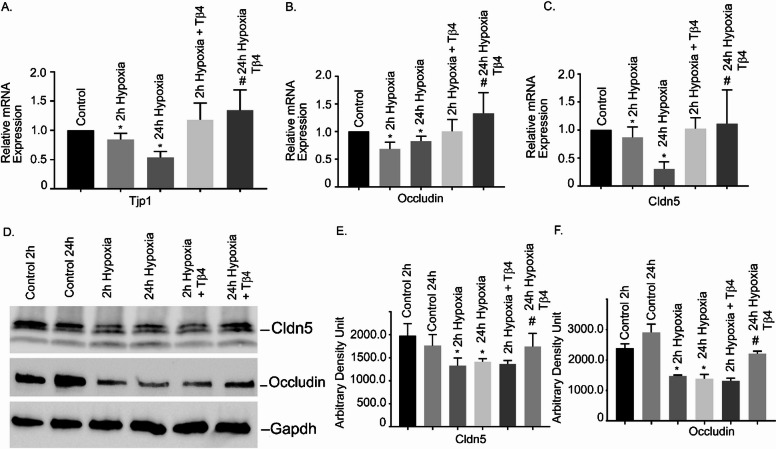
Effects of Tβ4 on hypoxia-induced alteration of TJ gene expression and protein levels in hBMVECs. hBMVECs were pretreated with Tβ4 (1 μg/mL) for 2 h and was subjected to 2 h and 24 h hypoxia. The mRNA expression of (**A**) Tjp1, (**B**) Occludin and (**C**) Cldn 5 were measured using reverse transcription-quantitative PCR. (**D**) Representative image of the Western blot analysis of Cldn5 and Occludin protein level. (**E**) The quantification of Cldn 5 and (**F**) Occludin of Western blot analyses. These experiments were replicated three-to-five times, and amplifications were performed and normalized to GAPDH. Results are presented as the mean ± SEM (*n* = 3–5). ^*^P < 0.05 vs. normoxia cells. ^#^*P* < 0.05 vs. hypoxia cells.

To determine the effects of initial and late hypoxia on the TJ gene expression, hBMVECs were exposed with 2 h and 24 h hypoxia. The result showed that both 2 h and 24 h hypoxia significantly decreased the mRNA expression of tight junction protein 1 (Tjp1), Occludin, and Claudin 5 (*P* < 0.05), compared with normoxic counterparts (Fig. 1A–C). Pretreatment with Tβ4 significantly restored their expressions (*P* < 0.05; Fig. 1A–C). Together, our data suggested that Tβ4 pre-treatment protected hypoxia-induced downregulation of TJ gene expression in hBMVECs.

Furthermore, we performed Western blot analyses to determine their protein level. As representative, we tested two critical TJ genes- Occludin and Claudin 5. The Western blot analyses showed that protein levels of both Occludin and Claudin 5 significantly reduced both at 2 h and 24 h hypoxia, compared to normoxic cells (Fig. 1D). The quantification of the Western analysis was shown in Fig. 1E, F, respectively. Pretreatment of Tβ4 resulted in moderate restoration of Occludin at 24 h treatment. We did not observe any restoration at 2 h treatment.

### Effects of Tβ4 on hypoxia-induced alteration of S1PR1 and SGPL1 gene expressions in hBMVECs

**Fig. 2:**
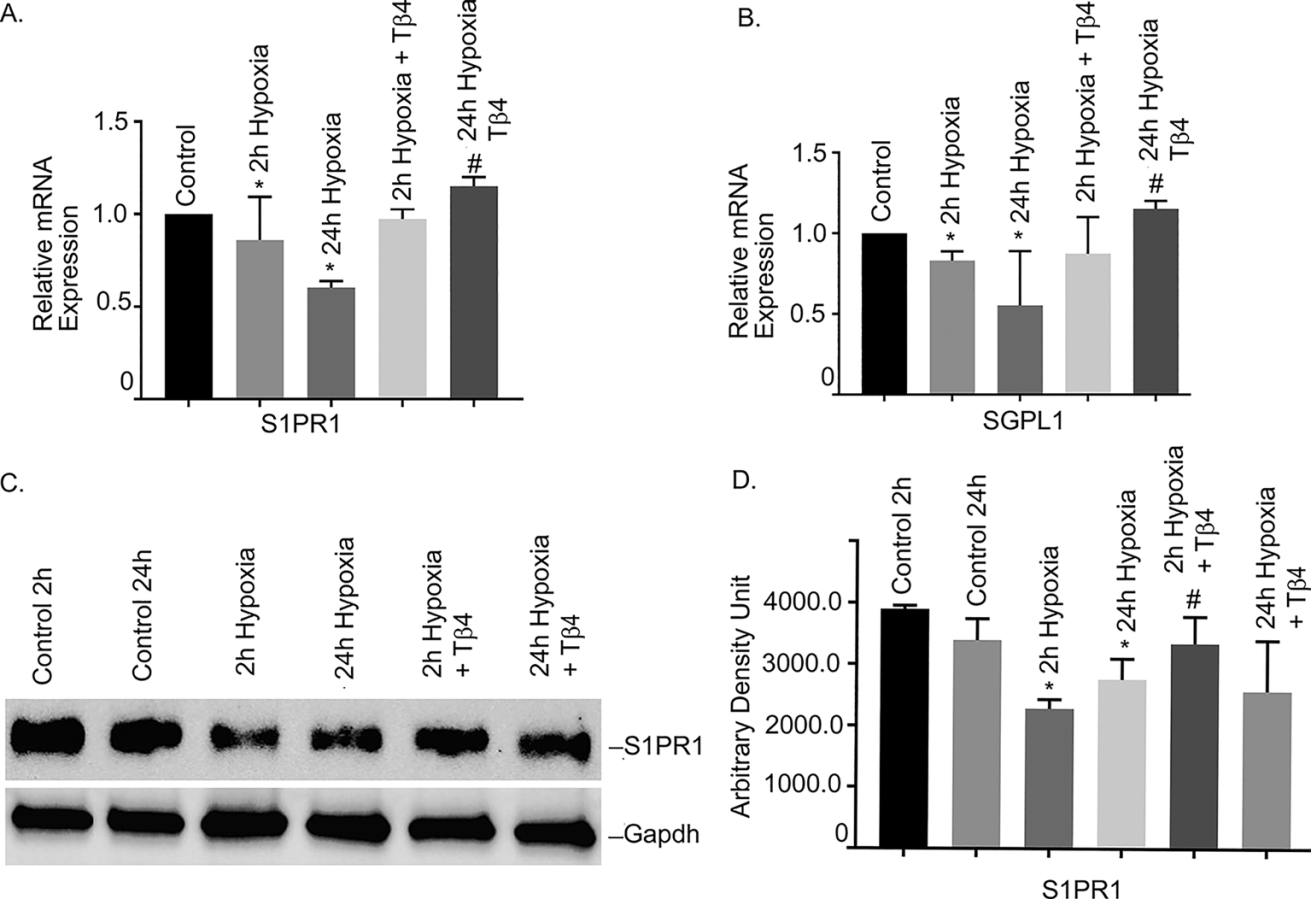

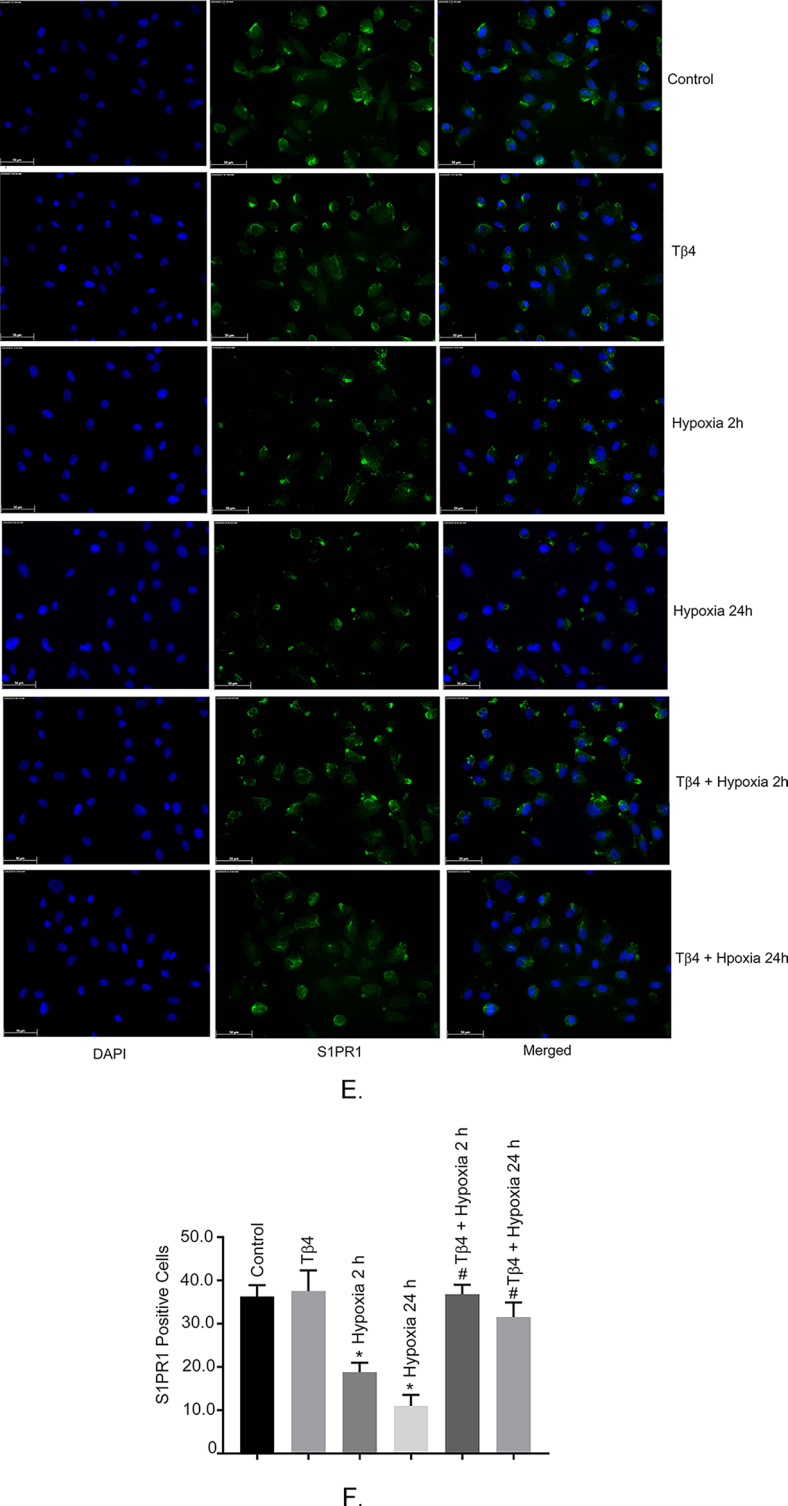
Effects of Tβ4 on hypoxia-induced alteration of S1PR1 and SGPL1 in hBMVECs. hBMVECs were pretreated with Tβ4 (1 μg/mL) for 2 h and was subjected to 2 h and 24 h hypoxia. The mRNA expression of (**A**) S1PR1 and (**B**) SGPL1 were measured using reverse transcription-quantitative PCR. (**C**) Representative image of the Western blot analysis of S1PR1 protein level. The experiments were replicated three times, and amplifications were performed and normalized to GAPDH. Results are presented as the mean ± SEM (*n* = 3). ^*^P < 0.05 vs. normoxia cells. ^#^*P* < 0.05 vs. hypoxia cells (**D**) The quantification of S1PR1 Western blot analysis (**E**) Cultured hBMVECs were pre-treated with Tβ4 for 2 h and then subjected to 2 h and 24 h hypoxia. (**F**) Proportion of S1PR1 green fluorescence positive cells are shown in graph. Three separate fields were used to count green fluorescence positive cells in each group. N = 3 microscopic fields/group. *P < 0.05 vs. normoxia cells, *P* < 0.05 vs. hypoxia cells. Dual staining immunofluorescence analysis of S1PR1 showed in green. Blue staining was DAPI, for the nucleus.

To examine the hypothesis that S1PR1 signaling is involved in hypoxia-induced BBB injury, we determined the mRNA expression of S1PR1 and SGPL1 in initial and late hypoxia (2 h and 24 h, respectively) in hBMVECs. Our data showed that both S1PR1 and SGPL1 mRNAs were significantly reduced under 2 h and 24 h hypoxia, compared to normoxic cells (Fig. 2, *P* < 0.05). To determine whether Tβ4 could reinstate their expression, data showed that Tβ4 pre-treatment significantly restored S1PR1 and SGPL1 expression (Fig. 2, *P* < 0.05). Our data suggested that Tβ4 may target S1P signaling and provided protection in hypoxia-induced vascular damaged in hBMVECs.

Additionally, we performed Western blot analysis to determine the protein level of S1PR1. The Western blot analysis showed that S1PR1 protein was significantly reduced both at 2 h and 24 h hypoxia, compared to normoxic cells (Fig. 2C and D). Pretreatment of Tβ4 showed significant restoration of S1PR1 protein in both conditions (*P* < 0.05).

Finally, under similar conditions, we evaluated S1PR1 level by immunofluorescence staining. We observed that hypoxia treatments (2 h and 24 h) significantly reduced S1PR1 protein levels as shown in Fig. 2E. However, hypoxia induced S1PR1 reduction was restored with pretreatment of Tβ4 as evidenced by reappearance of green fluorescence. The Proportion of S1PR1 green fluorescence positive cells are showing in Fig. 2F. Together, our data indicated that Tβ4 pre-treatment restored hypoxia induced S1PR1 reduction in hBMVECs.

### Effects of Tβ4 on hypoxia stimulated regulation of vascular cell adhesion molecule 1 (VCAM1), neuregulin1 (Nrg1), aquaporin 4 (AQ4) and BCRP in hBMVECs

**Fig. 3:**
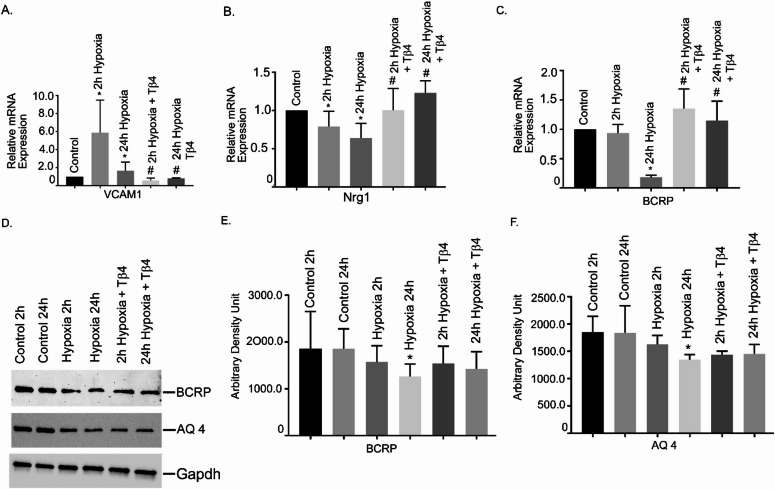
Effects of Tβ4 on hypoxia stimulated transcription regulation of vascular cell adhesion molecule 1 (VCAM1) and neuregulin1 (Nrg1), BCRP and AQ4 in hBMVECs. hBMVECs were pretreated with Tβ4 (1 μg/mL) for 2 h and was subjected to 2 h and 24 h hypoxia. The mRNA expression of (**A**) VCAM1 (**B**) Nrg1 and (**C**) BCRP were measured using reverse transcription-quantitative PCR. These experiments were replicated three times, and amplifications were performed and normalized to GAPDH. (**D**) Representative image of the Western blot analysis of BCRP and AQ4 protein level. (**E**) The quantification of BCRP and (**F**) AQ 4 of Western blot analyses. Results are presented as the mean ± SEM (*n* = 3). ^*^P < 0.05 vs. normoxia cells. ^#^*P* < 0.05 vs. hypoxia cells.

Adhesion molecules, like VCAM1 are critical in BBB integrity and are activated in BBB injury. Our study showed that VCAM1 was upregulated with 2 h and 24 h. (Fig. 3A, *P* < 0.05), compared to normoxic cells. Tβ4 pre-treatment significantly reduced the expression level (Fig. 3A, *P* < 0.05). Another molecule Nrg1, a neuroprotective factor was significantly reduced under 2 h and 24 h hypoxia and Tβ4 pre-treatment restored the level (Fig. 3B, *P* < 0.05). Our data suggested that Tβ4 may offer protection in hypoxia-induced vascular damaged in hBMVECs.

In addition to adhesion molecules, we examined the role of BCRP and AQ4 under hypoxia. Our data showed a significant reduction of BCRP gene expression and protein level in 24 h hypoxia, compared to normoxic cells. Tβ4 pre-treatment restored BCRP mRNA to the normal level (Fig. 3C, *P* < 0.05). Our data did not show any significant changes at 2 H hypoxia. However, at translational level, we observed reduction of both gene expressions at 2 h and 24 h hypoxia. Tβ4 pre-treatment partially restored BCRP protein both at 2 h and 24 h hypoxia (Fig. 3D, *P* < 0.05). We, next determine the water channel protein, AQ4. Our data showed significant reduction of AQ4 protein both at 2- and 24 h and Tβ4 pre-treatment partially restored the AQ level (Fig. 3D, *P* < 0.05). The quantification of BCRP and AQ 4 proteins are shown in Fig. 3E and F. Our data suggested that Tβ4 may offer protection in hypoxia-induced vascular injury and dysfunction of AQ4 and BCRP proteins in hBMVECs.

### Effects of Tβ4 on hypoxia-induced hBMVECs monolayer permeability and TEER assays

**Fig. 4:**
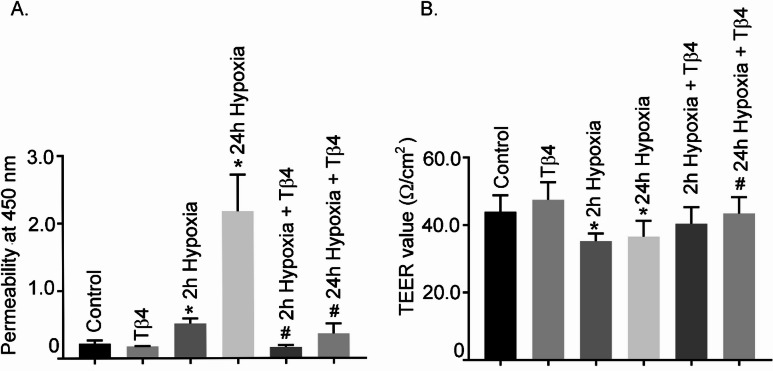
Effect of Tβ4 on hypoxia-induced hBMVEC monolayer permeability assay and TEER analysis. (**A**) Permeability assays were performed using Endothelial Trans-well Permeability Assay Kit (cat. No. CB6929, Cell Biologics, Inc.), according to manufacturer’s protocols. Data expressed as the A450 absorption readings, which are considered the relative permeability. Cultured hBMVECs were pre-treated with Tβ4 (1 μg/mL) for 2 h and subjected to 24 h hypoxia. Permeability assays were performed as described above. (**B**) Cultured hBMVECs were pre-treated with Tβ4 (1 μg/mL) for 2 h and subjected to 24 h hypoxia. The TEER analysis was performed using Millicell^®^ ERS-2 system. The TEER values are calculated as Ω/cm^2^. The permeability results are presented as the mean ± SEM (*n* = 3). ^*^P < 0.05 vs. normoxia cells. ^#^*P* < 0.05 vs. hypoxia cells. The TEER results are presented as the average ± SEM change of baseline TEER from at least three independent experiments consisting of at least three replicates.

To gain insight about Tβ4’s role in hypoxia-induced increased cellular permeability, hBMVECs were pre-treated with Tβ4 for 2 h before exposure to 24 h hypoxia. Hypoxia treatment significantly increased the permeability and was significantly reduced with prior treatment of Tβ4 suggesting a protective role in endothelial cell permeability (Fig. 4A). Under similar condition, the TEER assay was performed in hBMVEC monolayers. After treatment with hypoxia (oxygen 1%) for 2 and 24 h, the TEER in treated cells was significantly decreased as compared to the normoxic cells. Tβ4 pre-treatment under similar set-up showed improvement in the TEER values (Fig. 4B). This suggests a protective role of Tβ4 in endothelial cell permeability and barrier integrity during hypoxia-induced BBB impairment in hBMVEC.

### Effect of VPC23019 (a S1PR1 inhibitor) and Tβ4 on S1PR1 and SGPL1 gene expression in hBMVEC

**Fig. 5:**
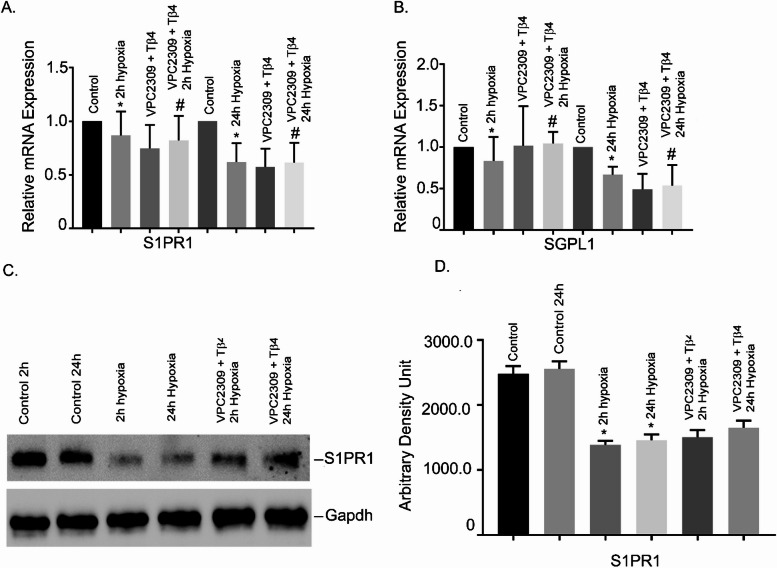
Effect of VPC23019 (a S1PR1 inhibitor) and Tβ4 on S1PR1 and SGPL1 gene expression in hBMVEC. hBMVECs were pretreated with VPC2309 (50 nmol) for 2 h followed by 2 h with Tβ4 (1 μg/mL) and then was exposed to 2 h and 24 h hypoxia. The mRNA expression of (**A**) S1PR1 and (**B**) SGPL1 were measured using reverse transcription-quantitative PCR. (**C**) Representative image of the Western blot analysis of S1PR1 protein level. (**D**) Quantification of S1PR1 Western blot analysis. Results are presented as the mean ± SEM (*n* = 3). ^*^P < 0.05 vs. normoxia cells. ^#^*P* < 0.05 vs. hypoxia cells.

To confirm whether S1PR1 is necessary in hypoxia induced TJ protein damage and Tβ4 targets S1PR1, hBMVECs were pre-treated with VPC23019 and Tβ4 followed by 2 h and 24 h hypoxia. The results showed that both S1PR1 and SGPL1 were significantly reduced at 2 h and 24 h hypoxia (Fig. 5A and B). It is interesting to observe that both normoxia and hypoxia groups treated for 2 h and 24 h, Tβ4 failed to restore their mRNA level back to normal (Fig. 5A and B).

We also performed Western blot analysis to determine the protein level of S1PR1. The Western blot analysis showed that S1PR1 protein was significantly reduced both at 2 h and 24 h hypoxia, compared to normoxic cells (Fig. 5C and D). Pretreatment of VPC23019 Tβ4 showed no significant restoration of S1PR1 protein under hypoxia conditions (*P* < 0.05).

### Effect of VPC23019 and Tβ4 on TJ gene expression in hBMVEC

**Fig. 6:**
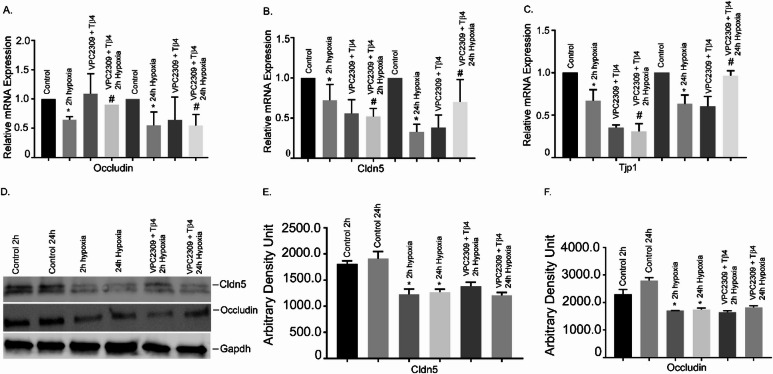
Effect of VPC23019 and Tβ4 on TJ genes expression in hBMVEC. hBMVECs were pretreated with VPC2309 (50 nmol) for 2 h followed by 2 h with Tβ4 (1 μg/mL) and then was exposed to 2 h and 24 h hypoxia. The mRNA expression of (**A**) Occludin, (**B**) Cldn 5 and (**C**) Tjp1 were measured using reverse transcription-quantitative PCR. (**D**) Representative image of the Western blot analysis of Cldn 5 and Occludin protein level. (**E**) The quantification of Cldn 5 and (**F**) Occludin Westerns were shown. These experiments were replicated three times, and amplifications were performed and normalized to GAPDH. Results are presented as the mean ± SEM (*n* = 3). ^*^P < 0.05 vs. normoxia cells. ^#^*P* < 0.05 vs. hypoxia cells.

To evaluate whether S1PR1 inhibition has any influence in TJ gene expression, hBMVECs were pre-treated with VPC23019 and Tβ4 followed by 2 h and 24 h hypoxia. The TJ genes, Occludin, Cldn5 and Tjp1 were analyzed. The result showed that all three TJ genes were significantly attenuated at 2 h and 24 h hypoxia, respectively (Fig. 6A-C). It is observed that in presence of VPC 23,019, Tβ4 failed to restore their mRNA level back to normal under the similar normoxia and hypoxia conditions (Fig. 6A-C). Together, our data suggests that S1PR1 is critical in BBB injury and Tβ4 may require S1PR1 for providing the protection.

We also performed Western blot analysis to determine the protein level of Occludin and Cldn5. The Western blot analysis showed that both Occludin and Cldn5 proteins were significantly reduced at 2 h and 24 h hypoxia, compared to normoxic cells (Fig. 6D and F). Pretreatment of Tβ4 showed no significant restoration of Occludin and Cldn5 protein under hypoxia (oxygen 1%) conditions (*P* < 0.05). The data suggested that S1PR1 is critical in BBB injury and Tβ4 may require S1PR1 for providing the protection of TJ proteins under hypoxia.

### Effect of VPC23019 and Tβ4 on hypoxia-induced hBMVECs monolayer permeability

**Fig. 7:**
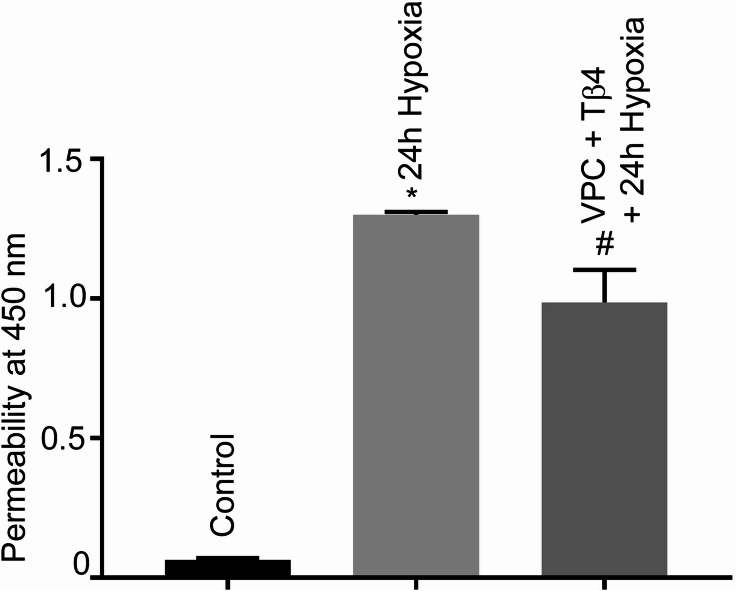
Effect of VPC23019 and Tβ4 on hypoxia-induced hBMVECs monolayer permeability. Permeability assays were performed as described in legend of Fig. 4A. Cultured hBMVECs were pre-treated with VPC2309 for 2 h followed by 2 h Tβ4 (1 μg/mL) and subjected to 24 h hypoxia. The permeability results are presented as the mean ± SEM (*n* = 3). ^*^P < 0.05 vs. normoxia cells. ^#^*P* < 0.05 vs. hypoxia cells. hBMVECs, human brain microvascular endothelial cells.

To get an insight about Tβ4’s role in hypoxia-induced increased cellular permeability, hBMVECs were pre-treated with VPC23019 and Tβ4 followed by 24 h of hypoxia. The data showed a significant increase in permeability under 24 h hypoxia (Fig. 7). However, cells treated with VPC23019 and Tβ4 did not show any significant reduction of permeability indicating that Tβ4 failed to provide protection and require S1PR1 as a partner to offer the protection in hypoxia-induced BBB damage.

## Discussion

The current study demonstrates that Tβ4 protects hypoxia-induced BBB from damage by targeting S1PR1 in cultured hBMVECs. We found that Tβ4 significantly rescued hypoxia-induced downregulation of TJ proteins, Nrg1, BCRP, AQ4, reduced hypoxia-induced hBMVEC permeability and reversed TEER by restoring S1PR1 level. The protective effect of Tβ4 on hypoxia induced BBB damage is S1PR1 dependent as evidenced by using selective inhibition of S1PR1 where Tβ4 failed to protect the hypoxia-induced BBB from damage.

The BBB is a specialized structure in the CNS and microvascular endothelial cells play a key role in healthy BBB function, acting as a molecular sheath and maintaining homeostasis of solute traffic. The selective solute dynamic is primarily controlled by endothelial TJ proteins which generate high endothelial electrical resistance and low paracellular permeability in the NVU. In hypoxic microenvironment like stroke, TBI, *etc*., blood supply is limited in BMVEC resulting excitotoxicity, inflammation and cell death that led to the loss of TJ protein in the NVU^41^. Downregulation of TJ proteins, particularly Claudin 5 and Occludin, are critical as they perform paracellular selective ion movement and maintain the electrical barrier integrity of NVU^42–44^. The possible reasons could be the involvement of hypoxia-induced transcription factor (HIFs) and rearrangement of cytoskeleton proteins *via* other TJ proteins like ZO-1^45,46^. In the present study, we observed significant decrease in mRNA expression of Tjp1, Occludin and Claudin 5 after 24 h hypoxia, however, a moderate level of reduction was noted in Tjp1 and Claudin 5 mRNA in 2 h hypoxia. The cellular pretreatment of Tβ4 was effective in promoting the expression of Tjp1, Occludin and Claudin 5. Furthermore, our data showed significant loss of Occludin and Claudin 5 protein level in 2 h and 24 h hypoxia and Tβ4 had restored them at 24 h hypoxia. A moderate level of restoration of Claudin 5 and Occludin were noted at 2 h hypoxia in Tβ4 treated cells. Finally, we showed that cellular permeability was significantly restored by Tβ4 treatment in presence of hypoxia, suggesting that the BBB was protected from damage when pre-treated with Tβ4. Overall, our goal of identifying the beneficial use and mechanisms of Tβ4 in attenuating the BBB damage we further suggest that the Tβ4 has a potent capability of restoring BBB damage under various stimuli that are common in traumatic brain injury (inflammatory & hypoxia). Previously, we have shown Tβ4 protected the LPS induced (inflammatory) BBB damage by restoring Claudin 5, Occludin, and Tjp1^29^. In the current study the hypoxia induced BBB damage (also a common occurrence in TBI) was again restored by Tβ4 via regulating S1PR1. The study further suggests that Tβ4 has the capability of restoring the BBB damage irrespective of the nature of stimulus.

In this study, we have provided a set of new genes whose function were unknow under hypoxia. Our data showed significant down regulation of Nrg1, BCRP and AQ4 genes under hypoxia. The role Nrg1 was first showed by our group previously in LPS-stimulated BBB damage^29^. Here, we also observed a similar pattern, an attenuation under hypoxia. The effect of hypoxia on BCRP and AQ4 were varied ^10–15,41,45,46^. Our study showed down regulation of both BCRP and AQ4 proteins under hypoxia and Tβ4 partially restored BCRP but not AQ4. Reduction of AQ4 may be explained with TBI. One study showed that AQ4 was reduced in the edematous cortex after 1 day TBI and water content was increased^47^. In culture astrocytes model, report suggested that knocking down of AQ4 protect against water influx during hypoxia^48^. A similar analogy can be drawn that reduction of AQ4 may reverse hypoxia-induced edema and improve brain. This is the first study that showed downregulation of AQ4 under hypoxia in BMVEC and provided an insight into potential therapeutic benefits. Our data showed significant reduction of BCRP mRNA and protein at 24 h hypoxia. The reduction of this ABC transporter, BCRP, in hypoxia may be explained its regulatory role at incorporation of various solutes and molecules at the cell membrane (BBB). Recently, it was reported that scaffold proteins such as moesin, radixin and ezrin are involved in regulating transport activity and function at BBB^49^. More studies are required to validate the observation in other models and warrant further investigation.

Another critical component of BBB damage is the reduction of electrical resistance, an EC barrier integrity. Our study demonstrated a significant increase in TEER activity with Tβ4 pre-treatment indicating that the BBB protected from injury. The mechanism by which Tβ4 offers protection is currently unknown. Tβ4 lacks secretory signal in the peptide structure, and no significant progress is established regarding any receptor mediated mechanism. It is an actin binding protein and actin is present in the cell surface and may speculate to anchor as a receptor^50^. Another study showed a putative receptor, Ku80, an ATP-dependent DNA helicase II, binds to Tβ4 and may serve as a biological vehicle for intracellular activities^51^.

S1P signaling primarily mediated by S1PR1 contributed a pivotal role in vascular stability, permeability, and several diseases including autoimmune disease and inflammation^52–54^. Furthermore, using endothelial specific *S1pr1* knockout mice, Yanagida et al. showed that S1PR1 controls the BBB integrity by regulating TJ and adhesion junction protein localization, suggesting a therapeutic avenue for neurological diseases^55^. In addition, there were reports which are associated with BBB protection in in vitro and in vivo rodent models^56,57^. Our study supported the view that hypoxia treatment significantly downregulated S1PR1 and SGPL1 mRNAs and S1PR1 proteins in BMVECs. Cells pretreated with Tβ4 rescued hypoxia-induced reduction of S1PR1 suggesting a protective role of Tβ4 mediated by S1PR1 signaling. Earlier studies conducted by various scientific groups have identified the protective role of Tβ4 in various other pathologies like colon cancer, cardiac and renal fibrosis, myocardial infarction, and skin diseases. Some of the Tβ4 regulatory signaling pathways identified under these approaches were the Wnt, Notch, PI3K/Akt/eNOS, Jak2/Stat3 and TGFβ/smad signaling pathways. Our current study is unique in its approach as for the first time Tβ4 has been evaluated for its efficacy in restoring hypoxia-induced BBB impairment via a newer mechanism of regulating the S1PR1 signaling. As mentioned earlier, S1PR1 is already a vital signaling molecule in some of the other human pathological conditions^23,26,58^ and our findings showing Tβ4 to be its regulatory molecule is quite novel. This is the first study demonstrating that Tβ4 is providing protection in hypoxia-induced BBB impairment by controlling S1P signaling.

To further validate S1PR1 being the target of Tβ4 and to delineate its role in BBB dysfunction we treated the cells with the S1PR1 antagonist (VPC23019) in the presence of Tβ4. Our data showed that Tβ4 failed to provide the protection in antagonist treated cells exposed to hypoxia and S1PR1 protein restoration. The transcription of both S1PR1 and SGPL1 were not restored in the antagonist and Tβ4 treated cells. Our data also showed that Tβ4 exhibited no restoration of other TJ proteins like Claudin 5 and Occludin in antagonist treated cells. It is possible that the antagonist did not completely inhibit these molecules and Tβ4 promoted the upregulation of these proteins. Importantly, the permeability assay showed that Tβ4 failed to provide the protection in presence of the antagonist suggesting S1PR1 is a target for Tβ4 for controlling BBB leakage. This is first report showing that S1PR1 is a target for Tβ4 in hypoxia-induced BBB dysfunction. Taken together, our data suggested that S1PR1 depletion may be key factor in hypoxia-induced BBB dysfunction and Tβ4-mediated S1PR1 activation is required to maintain BBB integrity in post hypoxia BBB damage.

Our findings cannot conclude that modulation of S1PR1 on BMVEC is the only mechanism responsible for the protection offered by Tβ4. Our study also identified AQ4, BCRP or Nrg1 as another set of candidates which played critical role in hypoxia. But the current study did not extend their role because of limitation and beyond scope. We particularly focused on S1PR1. S1PR1 is present in many other cell types which are involved in BBB damage and therefore other potential cellular targets must be identified beyond BMVECs. In addition to BMVEC, there are other cell types like pericytes, astrocytes and microglial cells which were also impacted by hypoxia and multiple biochemical pathways were activated. Therefore, an intricate physiological process is coordinated because of hypoxic insult which cannot be ignored. Specifically, astrocytes contribute a key role in effective functioning of BBB as BMVECs are surrounded by astrocytes end-feet^59^. Our study did not examine how astrocytes responded under hypoxia and the role of S1PR1 in astrocytes activation. This could be considered as a limitation of the current study. Regarding signaling the downstream pathways of S1PR1 such as Rac1 activation and actin cytoskeleton rearrangement which are key and affecting the barrier function. Rac is required to maintain integrity and normal barrier function while RhoA disrupts endothelial barrier function by enhancing the formation of stress fibers and focal adhesions ultimately maintains the barrier integrity and function. The current study focuses on Tβ4’s role in hypoxia-induced BBB disruption which is novel and the underlying mechanism of action of Tβ4 in BBB maintenance. We identified S1PR1 as a potential candidate which Tβ4 acted on and contributed its benefits in restoration of hypoxia-induced BBB injury. Therefore, we did not examine the downstream signaling mechanism in the current study and is beyond the scope of our current investigation. We plan to extend our signaling part and associated events in the next phase of study. The hBMVECs were chosen as the model system in the present study as it is one of the pivotal cells in the neurovascular unit. Therefore, the use of only hBMVECs is a limitation of the present study. Another limitation is that our study did not provide any in vivo data. This is important to understand Tβ4’s efficacy in a preclinical model like ischemia/stroke or hypoxic in vivo rodent model. The present study indicates a possible link between Tβ4 and S1PR1 for BBB restoration in hypoxic condition.

## Supplementary Information

Below is the link to the electronic supplementary material.

Supplementary Material 1

Supplementary Material 2

## Data Availability

The datasets used and/or analyzed during the current study are available from the corresponding authors on reasonable request.
