# Supplementary figures and images for: Thymosin β4 stabilizes hypoxia induced brain microvascular endothelial cell dysfunction through S1PR1 dependent mechanisms
Source: Sci Rep. 2025 Dec 1;15:45764. doi: 10.1038/s41598-025-28435-2 (PMC12756306; doi:10.1038/s41598-025-28435-2)

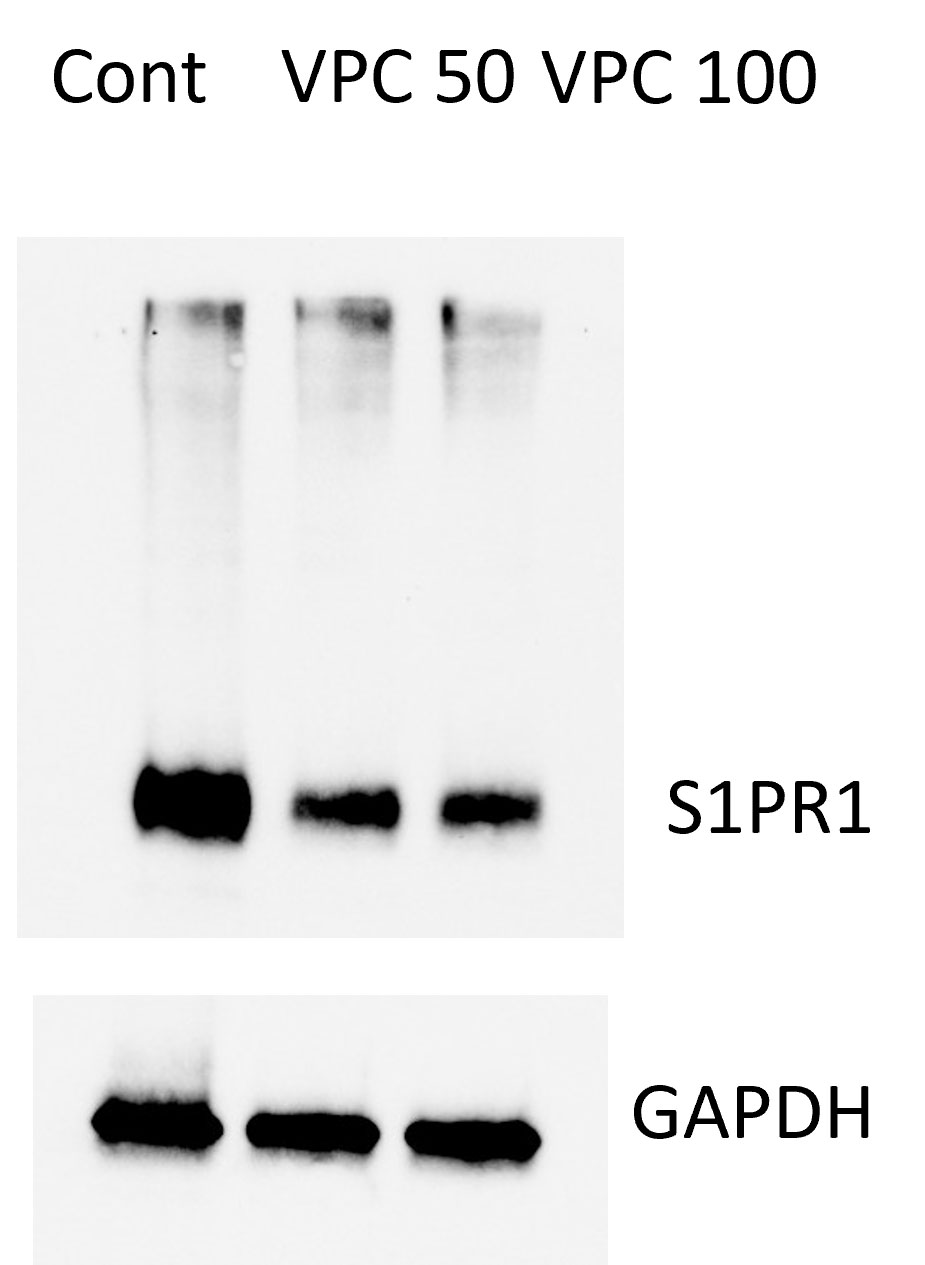

Supplement: Supplementary file 1 — Supplementary Material 1 [file 41598_2025_28435_MOESM1_ESM.jpg]
