# Supplementary material for: Thymosin β4 stabilizes hypoxia induced brain microvascular endothelial cell dysfunction through S1PR1 dependent mechanisms
Source: Sci Rep. 2025 Dec 1;15:45764. doi: 10.1038/s41598-025-28435-2 (PMC12756306; doi:10.1038/s41598-025-28435-2)
Supplement: Supplementary file 2 — Supplementary Material 2 [file 41598_2025_28435_MOESM2_ESM.pdf]

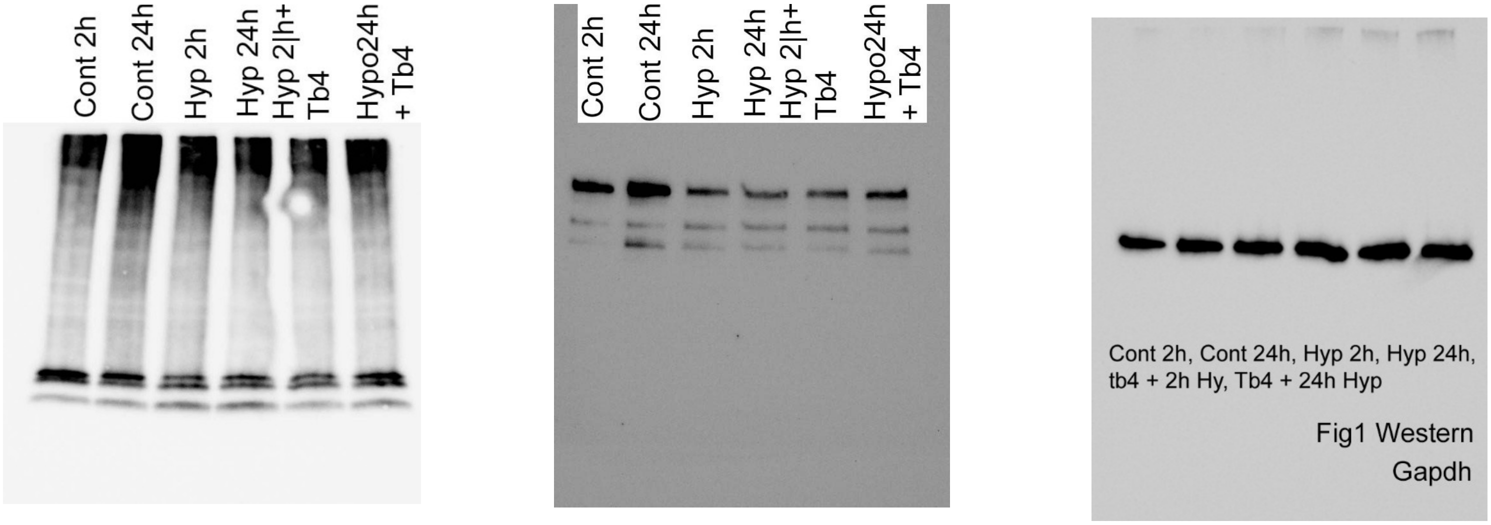

Fig1 Original uncropped blots for Cldn5, Occludin and Gapdh (Left to right)

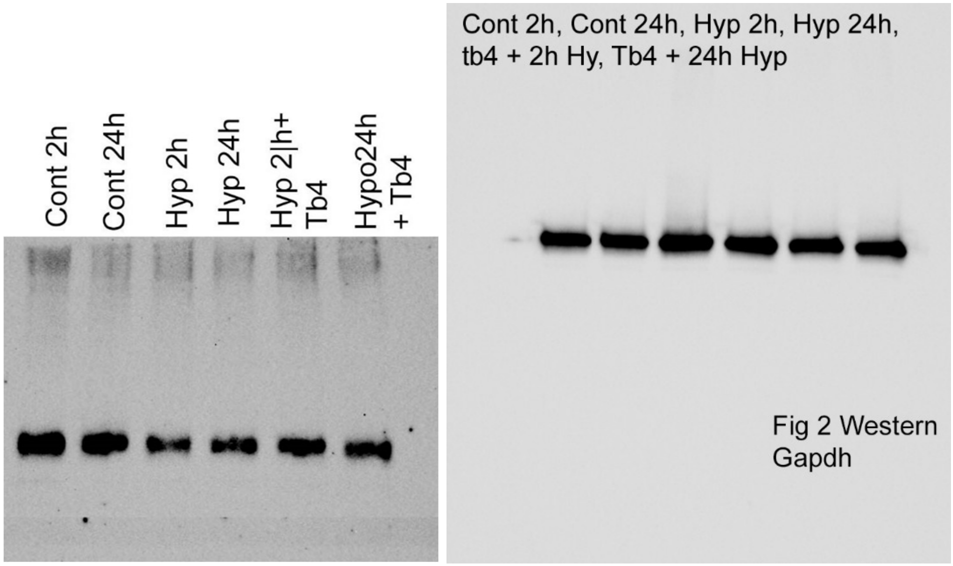

Fig 2 Original uncropped blots

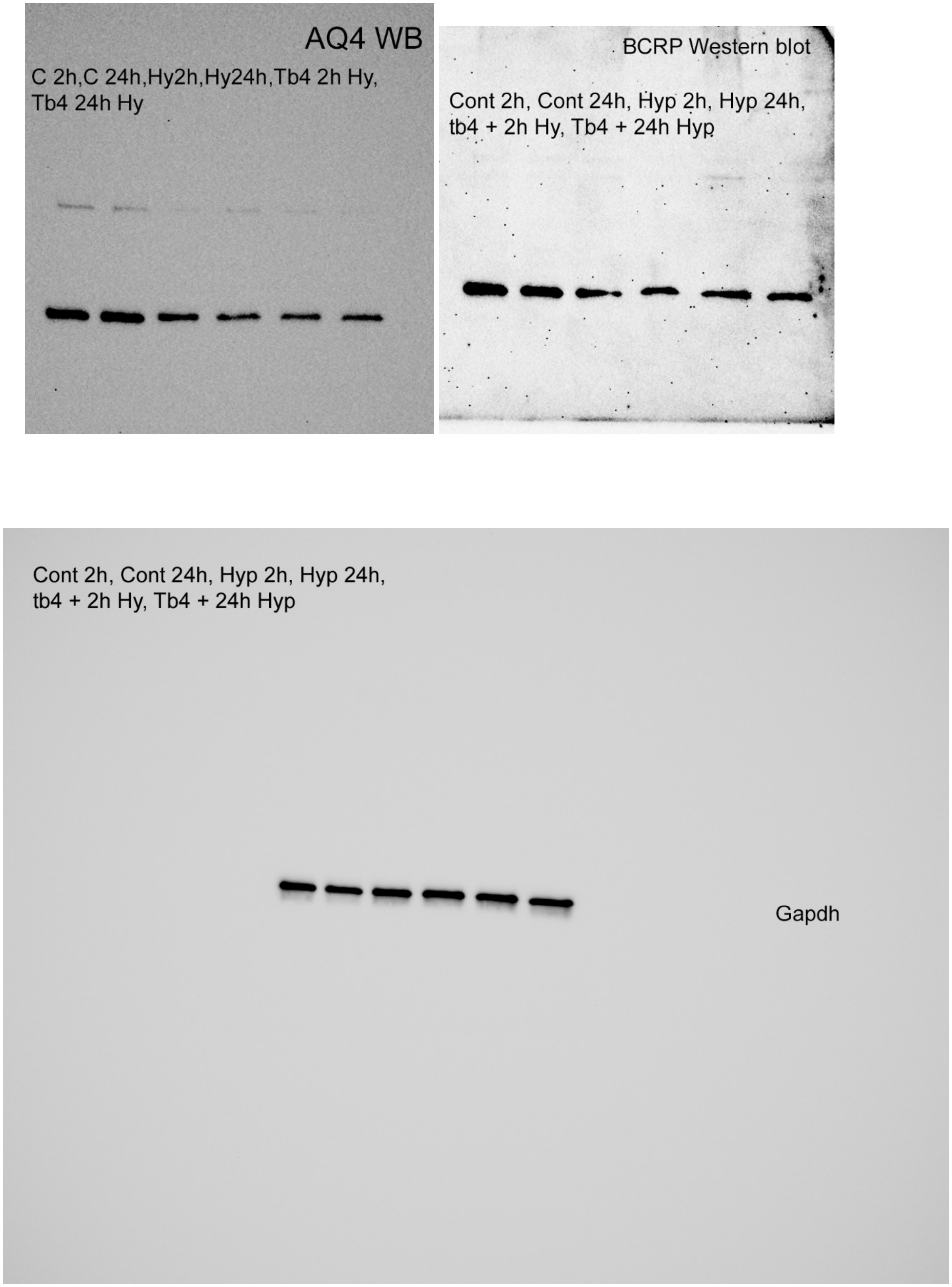

Fig 3. Original uncropped blots for AQ4 (upper left) and BCRP (upper Right). Gapdh is shown above.

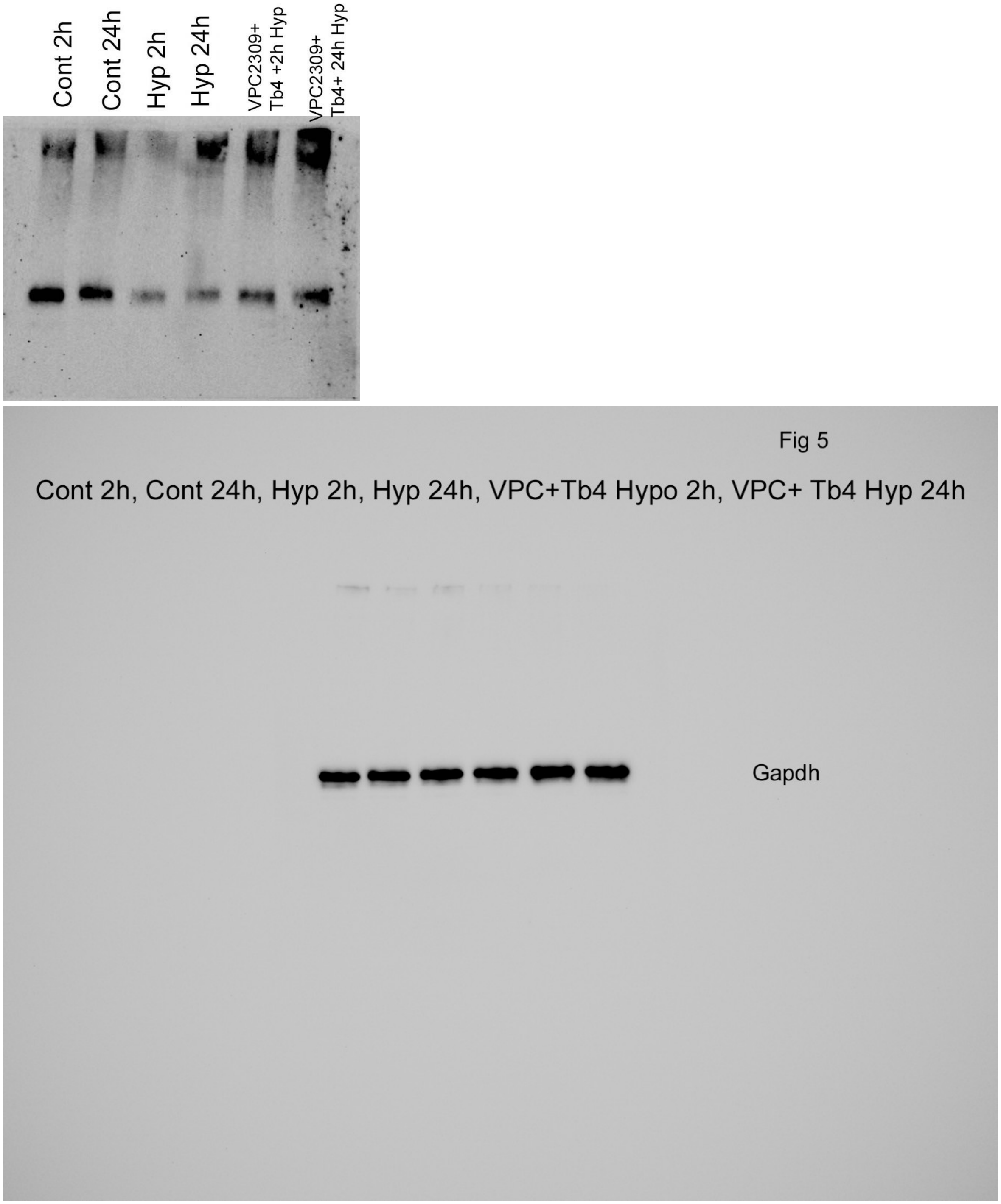

Fig5. Original uncropped blots for S1PR1 are shown in the upper panel and Gapdh is shown lower panel.

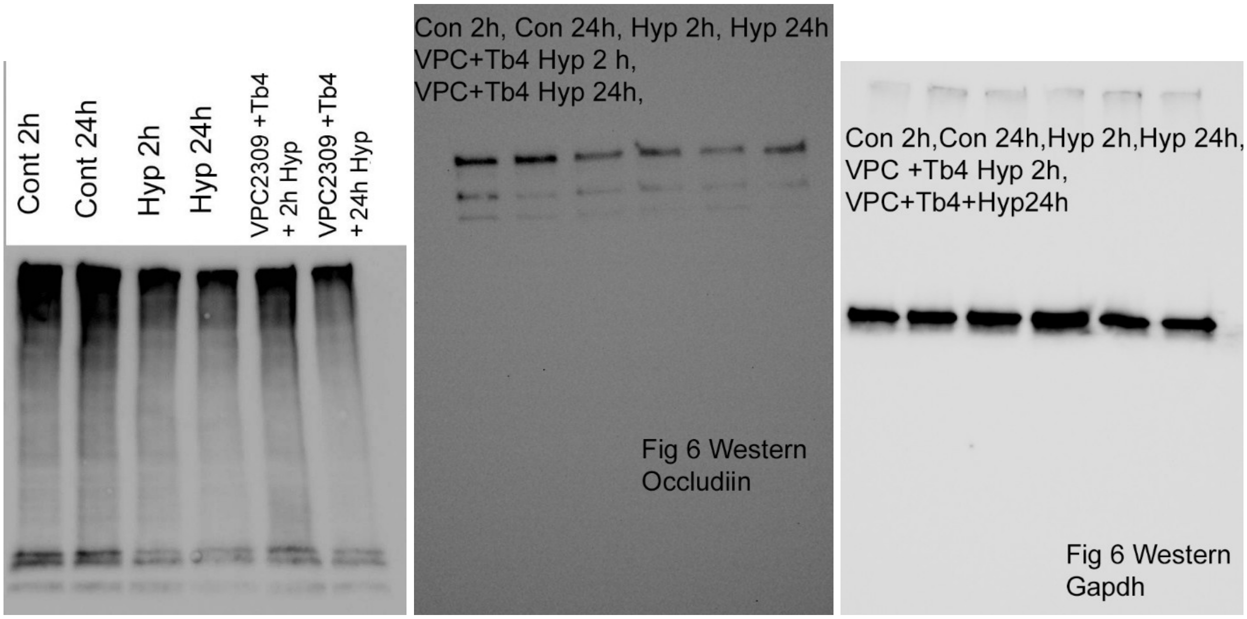

Fig 6. Original uncropped blots for Cldn5, Occludin and Gapdh are shown in the upper panel, left to right.
